# Exploring district nurses’ experiences with digital encounters in primary healthcare: a qualitative pilot study

**DOI:** 10.1186/s12875-026-03410-6

**Published:** 2026-06-06

**Authors:** Margaretha Larsson, Louise Friberger, Johanna Götvall, Irene Eriksson

**Affiliations:** 1 School of Health Sciences, University of Skövde, Skövde, Sweden; 2 Primary Healthcare Center, Hjo, Sweden; 3 Skövde, Sweden; 4 Department of Health Sciences, University West, Gustava Melins Gata 2, Trollhättan, S- 461 32 Sweden

**Keywords:** Primary healthcare, Qualitative research, Specialist nurse, Telehealth, Telemedicine

## Abstract

**Background:**

Primary healthcare (PHC) is often a person’s first contact with the healthcare system and they often meet district nurses. The need and demand for digital care encounters are increasing in healthcare services. The aim of this study was to highlight district nurses' experiences of working with digital care encounters in PHC.

**Methods:**

In this pilot study, a qualitative descriptive design was used to gain a deeper understanding of district nurses’ experiences. Data were analysed using qualitative content analysis according to Graneheim and Lundman [1].

**Results:**

The analysis resulted in one overarching theme and four categories. District nurses in PHC experienced digital patient encounters as complex, captured in the theme “complexity in navigating digital care in PHC.”, Four categories were identified: “digital care encounters entail opportunities”; “obstacles in digital care encounters”; “the impact of digital interaction on the patient encounter”; and “expectations and development needs in digital PHC.”

**Conclusion:**

Digital care encounters represent a new and complex aspect of district nursing that should be considered in implementation. Clearer routines and guidelines may support both nurses and patients in optimising digital care.

**Supplementary Information:**

The online version contains supplementary material available at 10.1186/s12875-026-03410-6.

## Background

The World Health Organization (WHO) has four guiding principles aimed at orienting the global strategy for the appropriate and sustainable use of digital health technology in national health sectors and national health strategies [2]. WHO also supports countries in prioritizing their national investment in digital health in support of PHC [2]. Globally, the adoption and integration of digital healthcare solutions vary considerably between countries, influenced by differences in healthcare infrastructure, policy, and resource allocation. Several international studies highlight both similar benefits—such as improved accessibility and patient engagement—and challenges, including digital literacy gaps and equity concerns, which align with experiences reported in Sweden [3]. The global population is getting older, which will in turn require a greater need for care and increased resources in the future [4].

PHC is often a person’s first contact with the healthcare system, and includes medical assessment and treatment, nursing, health promotion, and rehabilitation [5, 6]. It also guides patients within the healthcare system and coordinates care, offering greater freedom of choice than most other healthcare sectors. In Sweden, PHC is required to be easily accessible, person-centred, and to ensure good continuity of care [7]. Over the past decade, digital PHC visits have increased significantly [8, 9]. According to the Swedish National Board of Health and Welfare, digital healthcare services involve remote interactions via digital communication methods, including telephone, video, websites or mobile applications, between patients and healthcare professionals [10]. Expanding access to such services is seen as necessary to meet both societal digitalization trends and growing care needs [11]. Swedish patients can now independently book appointments via 1177.se, and demand for digital encounters is steadily increasing [12]. For district nurses, this means more frequent involvement in digital care delivery [13].

District nurses in Sweden receive training within the specialist nurse education program, which prepares them to work in both PHC and community home care settings. Similar to many other countries, Swedish specialist nurses are certified and hold a second-cycle higher education qualification, including a Master’s degree. The program is designed to strengthen nurses’ critical thinking, research competence, and ethical reasoning in both theoretical and clinical contexts [14, 15]. Ultimately, this education fosters advanced intellectual skills, enhances professional self-confidence [16], and supports career development [17]. Within their professional role, specialist nurses are also responsible for integrating digital tools into care, engaging patients, and promoting caring relationships—even in virtual settings [18–20]. This requires situation-specific sensitivity and skills adapted to the digital context [21, 22]. Person-centred care principles—including a holistic view of the patient’s physical, mental, social, and spiritual needs—remain central [23]. Continuity of care plays a crucial role in building trust and ensuring patients feel safe, and digital encounters can support this when integrated with, rather than replacing, physical visits [24–27]. Older patients, in particular, may be more willing to engage digitally when they have an established relationship with their district nurse, highlighting the complementary role of digital and in-person care [28].

Despite the potential benefits, district nurses in Sweden have reported mixed experiences with digital tools. While many view them as inevitable and beneficial, concerns remain about implementation, workload, and the preservation of person-centred care principles [29, 30]. Adapting to digital development may require expanding and redefining the district nurse role. They also consider it important to maintain central concepts within person-centered care in digitalized care visits [29].

This study explores district nurses’ experiences of working with digital care encounters in PHC, with a focus on how communication and caring relationships are affected in this form of patient contact.

## Methods

### Aim

The aim of this study was to highlight district nurses’ experiences working with digital care encounters in PHC.

### Design

In this pilot study, a qualitative descriptive design was used to gain a deeper understanding of district nurses’ experiences [31]. The study was designed as a pilot to explore the feasibility of the research approach, including recruitment procedures, data collection, and analysis, and to generate preliminary insights that could inform future larger-scale studies.

### Setting and participants

Initially, contact was made with the heads of 29 PHC centers through e-mail. Five heads agreed to participate and forwarded the requests and information about the study to district nurses employed in their centers. Of these 29 PHC centers, 15 had not yet started working with digital care encounters, five did not respond at all and four responded that they did not have the time or resources to participate. The inclusion criteria were participants who were educated district nurse and who had experience with digital care encounters. The district nurses who agreed to participate were contacted by the authors (LF, JG). They were then given additional verbal information about the purpose of the study, asked to participate in an interview, and were informed that the interviews would be recorded. Eight district nurses (*n* = 8) answered the request, and the eight nurses worked at five different PHC centers. Participants were aged 30–62 years and included two men and six women who had worked as district nurses in PHC for between two and 32 years and had between six months and three years of experience working in digital care encounters. Two of the participants had received half a day’s worth of education related to digital care encounters; however, all had received a brief overview of the system they were going to use. The district nurses were active in PHC centers located in small-to-medium–sized municipalities in southern and central Sweden.

### Data collection

**Fig. 1:**
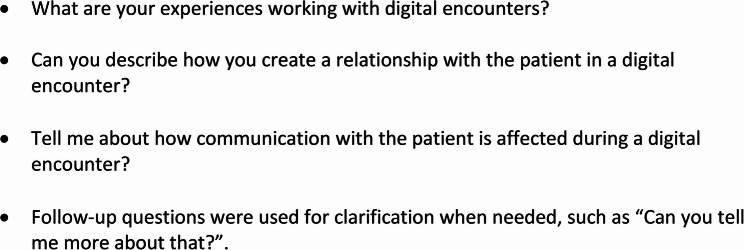
Interview guide

In this study, digital care encounters refer to video- and chat-based consultations, while telephone encounters were considered a separate modality. Data were collected through semi-structured interviews using an interview guide (Fig. 1). The guide covered participants’ experiences of digital care encounters, including perceived challenges, opportunities, and the impact on patient interactions. Follow-up questions were used to encourage participants to elaborate on their responses. Data collection was carried out by the second (LF) and third (JG) authors during February 2023. Interviews were completed in separate rooms in the district nurses’ workplaces or in their homes, lasted between 30 and 40 min, and were tape-recorded and transcribed verbatim.

### Ethical considerations

All steps in this pilot study were conducted following national ethical regulations and conform to the Declaration of Helsinki [1]. According to Swedish legislation, Swedish Ethical Review Authority approval was not needed for this study, since it did not include sensitive, personal data or affect participants’ health and well-being [32]. However, the study does comply with ethical standards for research, which means that the four ethical principles of respect for autonomy, beneficence, non-maleficence, and justice were considered throughout the study. The heads of five PHC centers approved participation in the study and forwarded the study request and information to district nurses who met the inclusion criteria. All participants received both verbal and written information about the study, the confidentiality of the data, and about their rights as participants, including their right to withdraw at any time without providing a reason. They then gave oral and written informed consent, and all data are reported at the group level.

### Data analysis

The data were analyzed using qualitative content analysis [33, 34]. All the authors read the interviews several times to confirm that they had a clear grasp of the whole. Meaning units describing the district nurses’ experiences of working in digital care encounters in PHC were identified. The meaning units were condensed into comprehensive units and then coded with labels describing the content. These labels were compared and those with similar meanings or which dealt with the same topics were grouped. Groups with similar meanings were then gathered to form categories, which were named with content-characteristic words, as described by Graneheim and Lundman [33] and Lindgren and Lundman [35]. All of the authors participated in the analysis process and discussed the final overarching theme and the four categories to achieve consensus. All of the authors participated in the analysis process; there was a constant back-and-forth movement between the entirety of the data material and the codes and themes derived during the analytical process. Quotes are provided in the findings for the purpose of illustration and validation. In interpreting the data, attention was paid to participants’ varying levels of training in digital healthcare, which ranged from brief introductions to more extensive education.

## Results

Content analysis of the interviews resulted in one theme: “complexity in navigating digital care in PHC” and four categories embodied therein, labelled: “digital care encounters entail opportunities,” “obstacles with the digital care encounters,” “the impact of digital interaction on the patient encounter,” and “expectations and development needs in digital PHC”. The theme and each of the categories are described in detail below.

### Complexity in navigating digital care in PHC

The district nurse perceived digital care encounters to be complex; intertwined with various opportunities, barriers, impacts, and expectations; and in need of further development. They viewed digital care as a supplementary form of PHC, with its strength lying in its ability to adapt to the patient’s needs. However, obstacles arose, as this form of care was not universally applicable to all patients or health issues. The digital interaction offered opportunities to gain insights into the patient’s daily life and the districts nurse’s own presence and work setting. The district nurse intuited multifaceted expectations to deliver digital care, in order to enhance the accessibility and availability of PHC for patients. Additionally, they highlighted the necessity of developing digital PHC to ensure patients comprehended which health issues were appropriate for digital care and could easily find bookable appointments.

### Digital care encounters entail opportunities

District nurses described digital care encounters as a positive delivery method that entailed several opportunities. By this, the district nurses meant that it was possible to adapt care encounters to the digital conditions and to the patient in focus. Digital care encounters were experienced as being more well-defined and focused on the purpose of the care encounter, compared to an encounter at a more traditional appointment. Digital care encounters were experienced as being short and efficient—without losing essential information and while maintaining the quality of care. District nurses often compared digital care encounters (e.g., video or chat) with traditional telephone consultations, highlighting differences in communication and patient interaction. Compared to a telephone encounter, digital care encounters were described as offering an extra dimension; the possibility to see patients on-screen facilitated assessments grounded in the patient’s situation. A district nurse narrates:

“Yes, it’s clear that you get another dimension when you see the patient, it’s never wrong. That is, you make your medical assessments based on what you see and hear and if you then get another sense, then it’s clearly easier to make a more correct assessment.” (Informant 6).

The district nurses stressed that the ability to see the patient via digital devices made the situation more personal and understandable. This was especially relevant when district nurses needed to see patients; for examples, during annual check-ups and follow-ups of patients with chronic diseases such as diabetes or hypertension or in case of mental illness as well as for assessments of eczema, allergies, bites, skin assessments, or rashes. The digital care encounter offered good conditions when it comes to working in a person-centered way, especially during follow-ups, check-ups, and when supporting patients. Both the patient’s narrative and seeing the patient’s appearances facilitated making an assessment. The district nurses considered that they had the ability to adapt the form of care based on the patient’s conditions. One district nurse described:

“Then they [patients] have prepared with blood pressure, height, and weight. All that is already ready before the visit and then it will be more like a check-up. Instead of having a visit that’s 45 minutes, I might have only a fifteen-minute visit! So, it really saves time while providing quality care.” (Informant 2).

The increased accessibility and flexibility that this form of patient contact provided was considered primarily beneficial for patients of working age, who then don’t need to take time off work and travel to the appointment. As one district nurse described, “The advantages are mainly for patients—they don’t have to take time off work… I have many who call from their workplaces…” (Informant 3). The district nurses considered digital care encounters as leading to fewer missed visits or cancelled visit due to illness, which led to fewer visits being rescheduled, and care appointments could take place despite illness, risk of infection, difficulties in getting to an appointment or patients being in a different location. The district nurses also mentioned that they saw advantages regarding the freedom to be able to work from home when necessary, such as when they themselves were ill and at risk of infecting others.

### Obstacles with the digital care encounter

District nurses described obstacles in digital care encounters, as these could not be used by all patients regardless of age. Some patients lacked a BankID and could not afford or did not have access to a computer or smartphone, which could lead to digital exclusion. Not every patient was adapted to digital care encounters, and whether they were depends on different personal conditions, such as attitudes and perceptions, as some patients preferred an encounter to be physical while others preferred them to be digital. The district nurses believed that PHC had to adapt to the individual and offer patients different contact routes and forms of encounters.

“It’s important that the right person is in the right place… I don’t think this will suit everyone… both when it comes to us working with it and the patients.” (Informant 3).

A common perception was that digital care encounters were not always appropriate, particularly when patients required physical assessment or specialized equipment, which necessitated rescheduling the appointment at a clinic or hospital. District nurses also expressed that when patients had not received or understood relevant information before booking a digital encounter, it often led to encounters being difficult to perform and not meeting patients’ expectations. In such cases, the digital format was experienced as unsuitable, and district nurses felt that their working hours were not being used effectively. One district nurse described:

“It has happened that people have applied for pain in the ear…and I can’t judge that online…and then we can’t do much…and then you have to refer them on, and it feels a bit silly.” (Informant 2).

The district nurses described that communication via digital screens could be one-dimensional and flat; for example, they missed possibilities to, take the patient by the hand, which they could do in a physical encounter. They stressed that it was difficult to discuss sensitive subjects in digital care encounters, especially in the case of established patient relationships. As one district nurse explained, “It can be more difficult to talk about sensitive issues in digital encounters. When you see each other, it sometimes feels more exposed, and there is also a sense of pressure to meet the patient’s expectations.” (Informant 5).

The district nurses sometime felt pressure and expectations related to not being able to meet the patient’s wishes. At a digital care encounter, these situations were more sensitive and difficult to handle.

Other obstacles that were described relate to difficulties with digital technology or poor internet connections, as reasons why digital care encounters became ineffective. The technology was deficient and could lead to poorer dialogue flow with patients, which made communication difficult. A good digital care encounter required that both patients and the district nurses were comfortable with the necessary technical and digital equipment. The district nurse stated that they were still unaccustomed to encountering patients in this way.

### The impact of digital interaction on the patient encounter

District nurses stated that being able to see the patient in their home environment or at work offered them a better picture of the patient as a person in comparison to a telephone call or a physical encounter.

“It’s like, now I see a person there, and I actually think I see the person in a different way. The focus becomes more on the person in that sense… and you also get a certain picture of how things are at home… or at work… when they come to the PHC, I have no idea about that.” (Informant 7).

The ability to read facial expressions and gestures contributed to better communication and improved relationships with the patients. Seeing the individual behind the voice helped them to create a picture of the patient from a person perspective. Accordingly, this made it easier to build a relationship with patients. On the other hand, in the case of already-established relationships between the patient and district nurse, digital care encounters were experienced as improving patient–provider relationships, as they provided opportunity for improved continuity, flexibility, and availability.

The district nurses described that encounter via digital devices contained less small talk, as they were more focused on the problem. This meant that patients’ opportunity to choose which form of contact they preferred can form the basis of a relationship. When patients chose to book digital care appointments, the district nurse assumed that they were requesting a quick assessment, which could result in satisfied patients and the creation of a relationship between a district nurse and a patient. A district nurse described:

“Those who contact me digitally aren’t looking for a long-term relationship or something that needs to be followed up like that…but it’s already a problem right there and then…then it will be more effective than a physical visit, you could say…but maybe it won’t be the same relationship, really.” (Informant 3).

The district nurses reflected over their own behavior during digital care encounters. Their experience was that they made a little more effort regarding their behavior, clothes, and posture as well as the importance of being calm. They also purported the importance of having a good working environment with the opportunity to maintain confidentiality during digital care encounters. The district nurses described feeling more recognized among patients, which was not always perceived positively.

### Expectations and development needs in digital PHC

The district nurses experienced offering digital care encounters as necessary to be competitive and believed expanding digitization in their work to be inevitable. They described digital care encounters as facilitating a strained care organization and as a complement to existing forms of care. For example, digital care encounters were experienced as taking less time compared to a physical visit; this enabled more bookable appointments in the long term, more assessments were carried out, and thus more patients had access to care.

“For us it can be… I’m speaking generally now, but healthcare often struggles with appointments and visits, and there never seem to be enough available, no matter how many there are. In that sense, though, I still view it positive.” (Informant 4).

They perceived expectations and pressure from patients, society, and employers to engage in digital care work. They stressed that healthcare was increasingly moving towards total accessibility, where patient could contact providers and have assess to care twenty-four hours seven days a week. The district nurses felt that patients expected them to offer digital care encounters, which facilitated patient’s everyday life in many aspects, and nurses felt that politicians (In Sweden) had decided to develop digital healthcare without actually evaluating how it should be done, and how it affects the quality of the care being provided. The district nurses meant that availability needed to increase to meet demands, but at the same time, they were worried about whether district nurses could or wanted to work these inconvenient working hours. One district nurse narrated:

“What I see…both as an advantage and a disadvantage is availability…many people want us to be available 24/7…I don’t want to be available around the clock.” (Informant 4).

District nurses stressed the need to develop digital care encounters in a patient-safe and efficient way for both patients and professionals. Information around digital care encounters needed increased clarity and guidelines so patients knew which types of bookings were suitable for a digital care encounter and what should be booked for physical assessment, or, alternatively, a telephone consultation appointment. They also purported that digital care marketing needed to be better, as not all patients knew that they offer digital care encounters and patients often do not find their digital services, which meant that the appointments were not used to their intended extent.

The district nurses stated that it was beneficial when patients were well-prepared for the digital care encounter and have been informed about the relevant conditions. It was optimal if the district nurses first had an opportunity to make an assessment of or triage the patient case and then independently book a digital care encounter.

## Discussion

The results from this pilot study highlight the intricate balance nurses must maintain when integrating digital care into PHC. It emphasizes the dual nature of digital care, where opportunities like enhanced adaptability and patient engagement coexist with challenges such as unequal access and the limitations of addressing certain health issues digitally. It underscores significant expectations placed on nurses to not only deliver effective digital care but also to guide patients in understanding its appropriate use. Ultimately, it calls for further development and support in digital health systems to ensure that the benefits of this care model can be fully realized, making it both accessible and effective for all patients. Of central importance for district nurses is to convey a sense of security and build relationships with patients, and video-based digital care encounters are perceived as more personal than telephone consultations, as they provide visual cues—an advantage also emphasized in previous research [27, 36]. This study reinforces the close connection between communication and relationship-building in nursing. The district nurses emphasized that establishing good contact with patients in digital encounters is essential, and that effective communication is a prerequisite for fostering trust and security. These findings align with previous research showing that effective nursing depends on strong relationships and that communication and relationship are inseparably linked [36, 37]. The findings indicate that digital care encounters are considered valuable for maintaining and even strengthening established patient–nurse relationships, particularly for patients with chronic diseases. Strong relationships are known to provide better conditions for accurate assessment [37], and when a connection already exists, regular digital communication can further reinforce this bond by supporting ongoing and consistent care [30]. Good communication skills create opportunities for person-centered practice [36], enabling nurses to see the individual beyond the disease. This becomes easier in digital encounters, where district nurses can better understand the patient through their own narration and by gaining insights into their home or work environment.

In other situations, the district nurses in this study report that sometimes it is less important to establish a deeper relationship with the patient; for example, when only quick contact and assessment are requested. It is considered that the digital care meeting is also well-suited for these types of care contacts, as it is more time-efficient while still providing a good quality of care. The experience is that it nevertheless contributes to a good relationship with patients, as these quick and efficient visits are in demand.

District nurses in this study emphasized the efficiency benefits of digital care encounters, particularly when used appropriately for routine follow-ups and minor health issues. This also relates to the concept of appropriateness in digital care, which is often clinician-centred. However, our findings suggest that patients also need support in understanding when digital care is suitable. These encounters promote greater accessibility and flexibility—both for patients, who avoid travel and can more easily fit consultations into daily life, and for nurses, who can manage care in a streamlined manner. Comparable findings in international research indicate that telemedicine and digital consultations are perceived as efficient, satisfactory, and beneficial for reducing unnecessary in-person visits while maintaining quality of care [38, 39].

Accessibility is a global issue [2], and Sweden’s municipalities and regions have started work in which increased accessibility within healthcare is the focus [40]. They purport that accessibility is lacking in many aspects, like need-based open hours, person-centered care, and the possibility of digital contact solutions and geographic proximity to PHC, as some examples [2, 40]. Ekman et al. [41] describe how care applications established in recent years have quickly become popular among the population who increasingly demand fast and, above all, easily accessible care. However, there is some ambivalence when it comes to how digitalization in PHC should be designed [27, 29, 30]. This is also confirmed in the results of the present study. District nurses express concern about how it will affect healthcare and how it will affect the working environment of district nurses in PHC in the future. At the same time, there is a certain concern and feeling that it is not always good that PHC is available all days all around the clock, as the results of this study show, and more could be expected from the district nurses’ availability, which may lead to changes to working hours. One reflection here is that healthcare itself may have contributed to society’s high expectations of accessibility, and that the more care becomes available, the more likely patients are to seek care. As a result, the population may become increasingly resource-demanding and care-seeking, and at the moment it is unclear whether expanded digital care encounters benefit this.

The results of this study show that the district nurses express frustration when digital care encounters were considered ineffective. When the patient encounter did not meet either the patient’s or the district nurse’s expectations, the district nurses express feelings of inadequacy, self-consciousness, and that the encounter had been unnecessary. The fact that the digital care encounter was not optimal could be due to various factors such as technical problems with sound or image as well as incorrect bookings, where the patient should have been assessed by another profession. Earlier research describes problems with digital care meetings when the technology does not work as it should, which negatively affects the meeting and its effectiveness [27, 42, 43]. Previous studies have also shown that in some cases communication between district nurse and the patient is difficult due to problems with sound and image, which negatively affects the experience of the encounter, and in some cases, the problem is so extensive that encounters have to be rescheduled [27, 42]. Nevertheless, in the present study, the experience is that using digital equipment usually works well and that both staff and patients are becoming increasingly comfortable and accustomed to using it, even within the older population. On the other hand, several studies have shown that many people do not have the necessary conditions to use digital care meetings due to factors such as age, disease, economic or social status, or language, which can create a digital exclusion [27, 43–45].

Although district nurses described challenges in navigating digital care in PHC, they emphasized its potential to enhance accessibility and efficiency. In line with earlier research, digital encounters are becoming increasingly accepted and comfortable, even among older patients [27, 28, 45]. Importantly, this study highlights that digital care is not intended to replace traditional encounters but should be regarded as a valuable complement that, when integrated thoughtfully, can optimize PHC.

The findings suggest that policies should focus on reducing digital exclusion through clearer patient information, improved booking visibility, and alternative care pathways. Targeted training for district nurses, alongside adequate resources and guidelines, could further enhance the quality, efficiency, and sustainability of digital PHC.

## Strengths and limitations

This pilot study was performed in a specific region of Sweden and is based on a small sample of participants, which may be a limitation of the study. A potential limitation is the recruitment strategy via PHC center heads, which may have introduced selection bias despite efforts to ensure variation across centers and regions. The relatively low sample size may also reflect the early stage of digital healthcare implementation, as well as time and resource constraints. Furthermore, the use of triangulation or member checking could have enhanced the credibility of the findings. The working method in digital encounters is in its infancy and that is probably why few have agreed to participate in this study. However, the interviews resulted in data that provided a deeper understanding, from both women’s and men’s perspectives, of district nurses’ experiences working with digital care encounters in PHC. The procedures and methods were presented as thoroughly as possible to strive for credibility and to make it possible for the reader to agree with and understand the logic of the findings. Furthermore, quotations were used to show that the findings were grounded in the interview texts in order to assure conformability. Potential interviewer bias, social desirability bias, and power dynamics between researchers and participants may have influenced the findings, despite efforts to minimize such effects. While qualitative results can never be universal, the knowledge may be transferable to similar PHC contexts in Sweden and countries with comparable systems; however, cross-country transferability may be limited due to structural and contextual differences [46]. We argue that findings from this pilot study can be useful for further development of digital care encounters in the PHC context.

## Conclusions

This pilot study contributes to a deeper understanding of digital encounters from the perspective of district nurses, which strengthens and confirms existing research in the field. The results verify that district nurses in PHC find it complex to work with digital care encounters, based on their profession, as it is a new form of work in traditional nursing, which needs to be considered during implementation. In this study, the complexity of safeguarding the caring relationship and the person-centered approach is described, as is how digital communication can affect this. Furthermore, opportunities can be seen to develop PHC that is more resource-saving, accessible, and at the same time, of good quality. Increased implementation of digital tools in PHC is considered inevitable and necessary to keep up with the digital development in society. Based on this, reflections and discussions can begin regarding the role of the district nurse within the profession and as part of the digital development in the future of PHC.

This study highlights what is central in digital patient encounters and how district nurses can relate, and work based on that. Suggestions and development areas are described regarding clearer routines and guidelines for patients and district nurses to optimize the use of digital care encounters. A continued need for research in the area is seen both from the perspective of patients and district nurses, as well as potential consequences when the implementation of digital care encounters in PHC is more established and advanced.

## Supplementary Information

Supplementary Material 1.

## Data Availability

Data is available on request.

## References

[1] World Medical Association. WMA Declaration of Helsinki: Ethical principles for medical research involving human participants. Jama. World Medical Association; 2024. 10.1001/jama.2024.21972 39425955

[2] WHO. Global strategy on digital health 2020–2025. World Health Organization; 2021.

[3] Erku D, Khatri R, Endalamaw A, Wolka E, Nigatu F, Zewdie, A, Assefa Y, et al. Digital health interventions to improve access to and quality of primary health care services: a scoping review. Int J Environ Res Public Health. 2023;20(19):6854. 10.3390/ijerph20196854. 10.3390/ijerph20196854 PMC10572344 37835125

[4] Graeden E, Bricker D. An ageing population needs a different approach to housing and care. World Economic Forum. 2022. https://www.weforum.org/stories/2022/10/ageing-population-care-housing-healthcare/.

[5] European Commission. State of Health in the EU Companion Repost 2021. 2022.

[6] Starfield B. Primary care: an increasingly important contributor to effectiveness, equity, and efficiency of health services. SESPAS report 2012. Gac Sanit. 2012;26(Suppl 1):20–6. 22265645 10.1016/j.gaceta.2011.10.009

[7] Swedish municipalities and regions. Agreement on Good and close care [in Swedish svenska kommuner och regioner, Överenskommelse om God och Nära vård]. 2024.

[8] VästKom. Annual Report 2023 [Årsrapport 2023]. Västsvenska kommunalförbundens samorganisation; 2023.

[9] Neves AL, Burgers J. Digital technologies in primary care: Implications for patient care and future research. Eur J Gen Pract. 2022;28(1):203–8. 35815445 10.1080/13814788.2022.2052041 PMC9278419

[10] National Boarder of Health and Welfare. Digital care service, overarching principles for care and treatment, [in Swedish, Digitala vårdtjänster – Principer för vilken vård och behandling som lämpar sig] 2018.

[11] Swedish municipalities and regions. Vision e-health 2025, [in Swedish Vision e-hälsa 2025]. Government Offices, Ministry of Social Affairs; 2020.

[12] 1177. 1177 Swedish healthcare direct. 2017.

[13] Hellzén O, Kjällman Alm A, Holmström Rising M. Primary Healthcare Nurses’ Views on Digital Healthcare Communication and Continuity of Care: A Deductive and Inductive Content Analysis. Nurs Rep. 2022;12(4):945–57. 36548164 10.3390/nursrep12040091 PMC9788199

[14] Dury C, Hall C, Danan JL, Mondoux J, Aguiar Barbieri-Figueiredo MC, Costa MAM, et al. Specialist nurse in Europe: education, regulation and role. Int Nurs Rev. 2014;61(4):454–62. 25214392 10.1111/inr.12123

[15] Wijk H, Öhlén J, Lidén E, Millberg LG, Jacobsson C, Söderberg S, Berg L, Engström Å, Höglund I, Lepp M, Lindström I, Nygren B, Person C, Petzäll K, Skär L, Suserud B-O, Söderlund M, et al. Verksamhetsförlagd utbildning på avancerad nivå — ny utmaning för specialistutbildningar för sjuksköterskor. Vård i Norden. 2009;29(4):41–3. 10.1177/010740830902900410.

[16] Watkins D. The influence of Masters education on the professional lives of British and German nurses and the further professionalization of nursing. J Adv Nurs. 2011;67(12):2605–14. 21615461 10.1111/j.1365-2648.2011.05698.x

[17] Pool IA, van Zundert H, Ten Cate O. Facilitating flexibility in postgraduate nursing education through entrustable professional activities to address nursing shortages and career prospects. Int Nurs Rev. 2024;71(3):419–23. 37822125 10.1111/inr.12892

[18] Isidori V, Diamanti F, Gios L, Malfatti G, Perini F, Nicolini A,Longhini J, Forti S, Fraschini F, Bizzarri G. Brancorsini S, Gaudino A. Digital technologies and the role of health care professionals: scoping review exploring nurses’ skills in the digital era and in the light of the COVID-19 pandemic. JMIR Nurs. 2022;5(1):e37631. 10.2196/37631. 36194466 10.2196/37631 PMC9579937

[19] Molina-Mula J, Gallo-Estrada J. Impact of Nurse-Patient Relationship on Quality of Care and Patient Autonomy in Decision-Making. Int J Environ Res Public Health. 2020;17(3). 10.3390/ijerph17030835 PMC7036952 32013108

[20] Swedish Nursing Association. Competence description for district nurses, [in Swedish Kompetensbeskrivning för distrktssköterskor]. 2019.

[21] Delmar C. The excesses of care: a matter of understanding the asymmetry of power. Nurs Philos. 2012;13(4):236–43. 22950727 10.1111/j.1466-769X.2012.00537.x

[22] Rosenlund M, Kinnunen UM, Saranto K. The Use of Digital Health Services Among Patients and Citizens Living at Home: Scoping Review. J Med Internet Res. 2023;25:e44711. 36972122 10.2196/44711 PMC10131924

[23] Jasemi M, Valizadeh L, Zamanzadeh V, Keogh B. A Concept Analysis of Holistic Care by Hybrid Model. Indian J Palliat Care. 2017;23(1):71–80. 28216867 10.4103/0973-1075.197960 PMC5294442

[24] Haggerty JL, Reid RJ, Freeman GK, Starfield BH, Adair CE, McKendry R. Continuity of care: a multidisciplinary review. BMJ. 2003;327(7425):1219–21. 14630762 10.1136/bmj.327.7425.1219 PMC274066

[25] Berntsson K, Eliasson M, Beckman L. Patient safety when receiving telephone advice in primary care – a Swedish qualitative interview study. BMC Nurs. 2022;21(1):24. 35042483 10.1186/s12912-021-00796-9 PMC8767717

[26] Gajarawala SN, Pelkowski JN. Telehealth Benefits and Barriers. J Nurse Pract. 2021;17(2):218–21. 33106751 10.1016/j.nurpra.2020.09.013 PMC7577680

[27] Donaghy E, Atherton H, Hammersley V, McNeilly H, Bikker A, Robbins L, et al. Acceptability, benefits, and challenges of video consulting: a qualitative study in primary care. Br J Gen Pract. 2019;69(686):e586–94. 31160368 10.3399/bjgp19X704141 PMC6617540

[28] Lindberg J, Bhatt R, Ferm A. Older people and rural eHealth: perceptions of caring relations and their effects on engagement in digital primary health care. Scand J Caring Sci. 2021;35(4):1322–31. 33448031 10.1111/scs.12953 PMC9290949

[29] Öberg U, Orre CJ, Isaksson U, Schimmer R, Larsson H, Hörnsten Å. Swedish primary healthcare nurses’ perceptions of using digital eHealth services in support of patient self-management. Scand J Caring Sci. 2018;32(2):961–70. 28960451 10.1111/scs.12534

[30] Entezarjou A, Bolmsjö BB, Calling S, Midlöv P, Milos Nymberg V. Experiences of digital communication with automated patient interviews and asynchronous chat in Swedish primary care: a qualitative study. BMJ open. 2020;10(7):e036585. 32709650 10.1136/bmjopen-2019-036585 PMC7380727

[31] Patton MQ. Qualitative research & evaluation methods: integrating theory and practice. 4th ed. Thousand Oaks: SAGE Publications, Inc.; 2015.

[32] The act on ethics review of research involving humans [in Swedish, Lagen om etikprövning av forskning som avser människor]. (SSF 2003:460).

[33] Graneheim UH, Lundman B. Qualitative content analysis in nursing research: concepts, procedures and measures to achieve trustworthiness. Nurse Educ Today. 2004;24(2):105–12. 10.1016/j.nedt.2003.10.001. 10.1016/j.nedt.2003.10.001 14769454

[34] Graneheim UH, Lindgren B-M, Lundman B. Methodological challenges in qualitative content analysis: A discussion paper. Nurse Educ Today. 2017;56:29–34. 10.1016/j.nedt.2017.06.002 28651100

[35] Lindgren BM, Lundman B, Graneheim UH. Abstraction and interpretation during the qualitative content analysis process. Int J Nurs Stud. 2020;108:103632. 32505813 10.1016/j.ijnurstu.2020.103632

[36] Höglander J, Holmström IK, Lövenmark A, Van Dulmen S, Eide H, Sundler AJ. Registered nurse-patient communication research: An integrative review for future directions in nursing research. J Adv Nurs. 2023;79(2):539–62. 36534429 10.1111/jan.15548

[37] Wiechula R, Conroy T, Kitson AL, Marshall RJ, Whitaker N, Rasmussen P. Umbrella review of the evidence: what factors influence the caring relationship between a nurse and patient? J Adv Nurs. 2016;72(4):723–34. 26692520 10.1111/jan.12862

[38] Gabrielsson-Järhult F, Kjellström S, Josefsson KA. Telemedicine consultations with physicians in Swedish primary care: a mixed methods study of users’ experiences and care patterns. Scand J Prim Health Care. 2021;39(2):204–13. 33974502 10.1080/02813432.2021.1913904 PMC8293950

[39] David OA, Costescu C, Cardos R, Mogoaşe C. How Effective are Serious Games for Promoting Mental Health and Health Behavioral Change in Children and Adolescents? A Systematic Review and Meta-analysis. Child Youth Care Forum. 2020;49(6):817–38.

[40] National Boarder of Health and Welfare. The plan to follow up and conduct dialouge about the accessibility of health care, [in Swdish Socialstyrelsens plan för att följa upp och föra dialog om hälso- och sjukvårdens tillgänglighet]. 2022.

[41] Ekman B, Thulesius H, Wilkens J, Lindgren A, Cronberg O, Arvidsson E. Utilization of digital primary care in Sweden: Descriptive analysis of claims data on demographics, socioeconomics, and diagnoses. Int J Med Inf. 2019;127:134–40. 10.1016/j.ijmedinf.2019.04.016 31128825

[42] Sturesson L, Groth K. Effects of the Digital Transformation: Qualitative Study on the Disturbances and Limitations of Using Video Visits in Outpatient Care. J Med Internet Res. 2018;20(6):e221. 29950290 10.2196/jmir.9866 PMC6041556

[43] Johansson AM, Lindberg I, Söderberg S. Healthcare personnel’s experiences using video consultation in primary healthcare in rural areas. Prim Health Care Res Dev. 2017;18(1):73–83. 27640522 10.1017/S1463423616000347

[44] Bertolazzi A, Quaglia V, Bongelli R. Barriers and facilitators to health technology adoption by older adults with chronic diseases: an integrative systematic review. BMC Public Health. 2024;24(1):506. 38365698 10.1186/s12889-024-18036-5 PMC10873991

[45] Thiyagarajan A, Grant C, Griffiths F, Atherton H. Exploring patients’ and clinicians’ experiences of video consultations in primary care: a systematic scoping review. BJGP Open. 2020;4(1). 10.3399/bjgpopen20X101020 PMC7330183 32184212

[46] Dahlberg K, Dahlberg H, Nyström M. Reflective lifeworld research. 2:1 ed. Stockholm: Studentlitteratur; 2008.

