# Supplementary material for: Exploring district nurses’ experiences with digital encounters in primary healthcare: a qualitative pilot study
Source: BMC Prim Care. 2026 Jun 6;27:226. doi: 10.1186/s12875-026-03410-6 (PMC13242665; doi:10.1186/s12875-026-03410-6)
Supplement: Supplementary file 1 — Supplementary Material 1. [file 12875_2026_3410_MOESM1_ESM.docx]

Figure 1. interview guide

- What are your experiences working with digital encounters?
- Can you describe how you create a relationship with the patient in a digital encounter?
- Tell me about how communication with the patient is affected during a digital encounter?
- Follow-up questions were used for clarification when needed, such as “Can you tell me more about that?”.
